# Biofilm-derived *Legionella pneumophila* evades the innate immune response in macrophages

**DOI:** 10.3389/fcimb.2013.00018

**Published:** 2013-05-27

**Authors:** Arwa Abu Khweek, Natalia S. Fernández Dávila, Kyle Caution, Anwari Akhter, Basant A. Abdulrahman, Mia Tazi, Hoda Hassan, Laura A. Novotny, Lauren O. Bakaletz, Amal O. Amer

**Affiliations:** ^1^Department of Microbial Infection and Immunity, Center for Microbial Interface Biology, College of Medicine, The Ohio State UniversityColumbus, OH, USA; ^2^Department of Biochemistry and Molecular Biology, Faculty of Pharmacy, Helwan UniversityCairo, Egypt; ^3^Center for Microbial Pathogenesis, Nationwide Children's HospitalColumbus, OH, USA

**Keywords:** biofilm, inflammasome, flagellin, caspase-1, *Legionella pneumophila*, innate immunity

## Abstract

*Legionella pneumophila*, the causative agent of Legionnaire's disease, replicates in human alveolar macrophages to establish infection. There is no human-to-human transmission and the main source of infection is *L. pneumophila* biofilms established in air conditioners, water fountains, and hospital equipments. The biofilm structure provides protection to the organism from disinfectants and antibacterial agents. *L. pneumophila* infection in humans is characterized by a subtle initial immune response, giving time for the organism to establish infection before the patient succumbs to pneumonia. Planktonic *L. pneumophila* elicits a strong immune response in murine, but not in human macrophages enabling control of the infection. Interactions between planktonic *L. pneumophila* and murine or human macrophages have been studied for years, yet the interface between biofilm-derived *L. pneumophila* and macrophages has not been explored. Here, we demonstrate that biofilm-derived *L. pneumophila* replicates significantly more in murine macrophages than planktonic bacteria. In contrast to planktonic *L. pneumophila*, biofilm-derived *L. pneumophila* lacks flagellin expression, do not activate caspase-1 or -7 and trigger less cell death. In addition, while planktonic *L. pneumophila* is promptly delivered to lysosomes for degradation, most biofilm-derived bacteria were enclosed in a vacuole that did not fuse with lysosomes in murine macrophages. This study advances our understanding of the innate immune response to biofilm-derived *L. pneumophila* and closely reproduces the natural mode of infection in human.

## Introduction

*Legionella pneumophila* (*L. pneumophila*) is a Gram negative facultative bacterium with fastidious growth requirements. Although *Legionella* exists as free-living planktonic forms in the environment, they are more commonly found as intracellular parasites of protozoans such as *Acanthamoeba* spp., *Hartmannella* spp., and *Tetrahymena* spp. (Atlas, 1999; Brown and Barker, 1999) and as inhabitants of mixed-community biofilms (Rogers et al., 1994; Lau and Ashbolt, 2009). Replication of *L. pneumophila* within amoeba is utilized as a survival strategy to overcome the low-nutrient environment and increases the resistance to disinfectant (Lau and Ashbolt, 2009). This opportunistic pathogen most often thrives in bacterial communities encased in extracellular polymeric matrix known as biofilm (Costerton et al., 1978; Donlan et al., 2005). Biofilms have been recognized as one of the most important factors of survival and proliferation of *L. pneumophila* in warm, humid environments like showers, air conditioners, and spa baths (Fraser et al., 1979; Fliermans et al., 1981; Sethi and Brandis, 1983; Spitalny et al., 1984; Abu Kwaik et al., 1993; Lettinga et al., 2002). These communities have been identified as a causative source of infection in susceptible hosts who inhale aerosols of contaminated water containing *L. pneumophila*. In the human lung environment, *L. pneumophila* replicates exponentially within alveolar macrophages prior to lysing the host cell and invading other macrophages causing a type of walking pneumonia called Legionnaire's disease or Legionellosis (Horwitz and Silverstein, 1980; Harb and Abu Kwaik, 2000). Legionellosis has two clinically distinct forms: Legionnaires' disease, a severe type of infection, which includes pneumonia and Pontiac fever, a milder self-limiting illness (Lau and Ashbolt, 2009). Approximately 20,000 cases of Legionnaire's disease are reported yearly in the US with no person-to-person transmission (Marston et al., 1997). Thus, using biofilm-derived *L. pneumophila* to study the innate immune response to infection recapitulates natural mode of infection in human.

The murine innate immune response to planktonic *L. pneumophila* has been studied extensively. Nlrc4 and Naip5 detect flagellin monomers in the host cytosol in a process that is dependent upon a functional bacterial type IV secretion system. The sensing of contaminating molecules of flagellin promotes the formation of a multi-protein complex called the inflammasome. Within the inflammasome, caspase-7 is activated downstream of caspase-1 which results in bacterial restriction via fusion of *L. pneumophila*-containing vacuoles with lysosomes (Coers et al., 2000; Akhter et al., 2009; Amer, 2010). Conversely, human monocytes do not activate this response upon *L. pneumophila* infection and phagosomes containing *L. pneumophila* evade fusion with the lysosome allowing bacterial replication (Roy, 2002; Isberg et al., 2009).

Here we demonstrate that biofilm-derived *L. pneumophila* replicates significantly more than planktonic *L. pneumophila* in murine macrophages due to diminished flagellin expression. Biofilm-derived *L. pneumophila* does not activate caspase-1 or caspase-7, evades fusion with lysosomes, and promotes less cell death. Taken together, our study characterizes the innate immune response to biofilm-derived *L. pneumophila* in murine and human macrophages.

## Methods

### Bacterial strains

*L. pneumophila* strain JR32 a wild-type (WT) strain and *flaA* mutant is deficient in flagellin, were kindly provided by Dr. Howard Shuman, University of Chicago. The *dotA* mutant, a JR32-derived strain defective in the Dot/Icm Type IV secretion system was kindly provided by Dr. Craig Roy, Yale School of Medicine. *L. pneumophila* expressing green fluorescent protein (GFP) was used for microscopy.

### *L. pneumophila* growth

*L. pneumophila* strains were grown on buffered charcoal yeast extract (BCYE) plates at 37°C. Three days later, the bacteria were resuspended in 5 ml of *L. pneumophila* medium (BYE) with additives (ferric nitrate, L-cysteine, thymidine) and vortexed 100× at high speed. For biofilm formation, a bacterial suspension with an optical density (OD) at 600 nm of 3.5 was diluted to 1:2500 in supplemented broth and 200 μl of this suspension was inoculated into each well of an 8-well chamber slide (Thermo Scientific Lab Tek chambered coverglass with cover #155411 and/or #177402). Slides were incubated at 37°C, 5% CO_2_ incubator with humidified atmosphere without shaking. Biofilms were fed by delivery of fresh medium to one side of the chamber slide well every 24 h for 6 days using 100 μl of *L. pneumophila* medium. The OD values were 3.4–3.6 and 3.8–4 for the JR32 and *dotA* mutant, respectively. *L. pneumophila* was grown for planktonic culture as previously described (Amer et al., 2006; Akhter et al., 2009, 2012).

### Macrophage infection

C57BL/6 mice were purchased from Jackson laboratory. Bone marrow derived macrophages (BMDMs) were prepared from the femurs of 6 to 8-week-old mice as previously described (Akhter et al., 2009, 2012). Isolation and preparation of the human monocyte-derived macrophages (hMDMs) from peripheral blood was carried out as previously described (Santic et al., 2005; Al-Khodor et al., 2008). Planktonic infections were used from post-exponential cultures as previously described (Amer et al., 2006; Akhter et al., 2009). Infection from biofilm derived *L. pneumophila* was carried as follows. Briefly, on day seven, the media was aspirated and transferred to a 50 mL tube. Biofilms were scraped from the chamber slide wells. Chambers were washed 2× with 200 μl of fresh LP medium with the previously mentioned additives and drained into the 50 ml tube. The collected biofilms were vortexed 100× and the OD at 600 nm of the collected suspension was used to calculate the desired multiplicity of infection. Equivalent inocula of planktonic bacteria were used for infection (MOIs).

*L. pneumophila* is an intracellular pathogen that replicates only within eukaryotic cells since the culture media do not contain required nutrients such as iron and cysteine.

### Confocal laser scanning microscope visualization

On day seven, biofilms were washed gently with 200 μl of sterile saline (0.9% sodium chloride) (Hospira 0409-4888-10), and stained using the Live/Dead BacLight Bacterial Viability Kit (Invitrogen #7007) for 15 min at room temperature protected from the light. Wells were washed 2× with sterile saline and 200 μl of 10% formalin was added for 24 h to fix the biofilm and stored at room temperature protected from the light with before it was visualized using inverted confocal Zeiss LSM 510 META microscope with a 63× water objective. Z-stacks were captured every 1 μm.

### Enzyme-linked immunosorbent assay (ELISA)

*L. pneumophila* JR32 and *dotA* strains from post-exponential planktonic and biofilm cultures were used to infect murine macrophages for 24 h. Supernatants were collected and centrifuged at 1200 rpm for 10 min and stored at −80°C as previously described (Abdulrahman et al., 2011). The plates were coated with primary antibody for IL-1β and ELISA were performed according to the manufacture specifications (R&D) (Abdulrahman et al., 2011).

### Western blot

Macrophage lysates were prepared following infection with either planktonic or biofilm JR32 or *dotA* mutant and immunoblotted with caspase-1, caspase-7, or β-actin antibodies (caspase-1, 1:3000; caspase-7, 1:300). Blots were washed and the corresponding secondary antibody was added. For flagellin detection by western blot, one OD of bacterial culture was pelleted and resuspended in SDS-containing sample buffer from planktonic or biofilm grown bacteria. Eighteen μ l were loaded on 12% SDS-PAGE gel. The blot was probed with flagellin antibody (1:100) kindly provided by Dr. Howard Shuman, University of Chicago, followed by the secondary antibody, donkey anti-rabbit (1:5000). Blots were developed after adding ECL Western Blotting Detection Reagent (GE Healthcare Amersham).

### Macrophage cytotoxicity assay

Percentage of macrophage necrosis was determined by measuring the release of host cell cytoplasmic lactate dehydrogenase (LDH) using the cytotoxicity detection kit (Roche Applied Science) to the specification of the manufacturer. BMDMs were infected with JR32 or the *dotA* mutant from either planktonic or biofilm culture for 4 or 24 h at an MOI of 0.5. Supernatants were collected and LDH release was calculated as previously described (Abdulrahman et al., 2011).

### *L. pneumophila* colocalization with lysotracker

*L. pneumophila* JR32 from post-exponential planktonic or biofilm cultures were used to infect macrophages plated in 24-well plates containing sterilized coverslips. Lysotracker red (1:500) was added before fixation and 4′, 6-diamidino-2-phenylindole (DAPI) was added after fixation. Coverslips were mounted on slides and viewed using the Olympus Flow View FV10i CLSM. Three hundred bacteria were counted from 2 coverslips for each condition.

### Contact-dependent hemolysis

Sheep RBCs (sRBCs) were diluted in RPMI, and washed 3× by centrifugation for 10 min at 2000× g until the supernatant did not show any signs of hemolysis; the cells were counted using a hemo-cytometer chamber. Reactions were set up in a final volume of 1 ml with a final concentration of 1 × 10^7^ sRBCs/ml. The sRBCs were incubated with the planktonic or biofilm bacteria at an MOI of 20 and RBC lysis was determined as previously described (Kirby et al., 1998; Alli et al., 2000).

### Scanning electron microscopy (SEM)

*L. pneumophila* strains were grown on 12-well plate coverslips for seven days. On the seventh day, the medium was aspirated and the coverslips were washed with 1× DPBS. Coverslips were fixed with 2.5% gluteraldehyde in 0.1 M phosphate buffer pH 7.4, processed and viewed by SEM.

### Statistical analysis

Experiments were performed 2–3 independent times each in triplicate or quadruplicate and yielded similar results. Comparisons of groups for statistical significance were performed using Student's tow tailed *t*-test. *P*-values ≤ was considered significant.

## Results

### The Dot/Icm type IV secretion system promotes robust *L. pneumophila* biofilm formation

To reproduce biofilm formation *in vitro*, WT *L. pneumophila* (JR32) and *dotA* mutant were grown for 7 days at 37°C on 5% CO_2_ in 8-well chambered coverslips and fed with *L. pneumophila* BYE media every 24 h. On the seventh day, biofilms were stained with Live/Dead stain then observed by confocal microscopy. *L. pneumophila* JR32 produced a thick biofilm with a maximum height of ~120 μm and exhibited filamentous structures.

**Figure 1:**
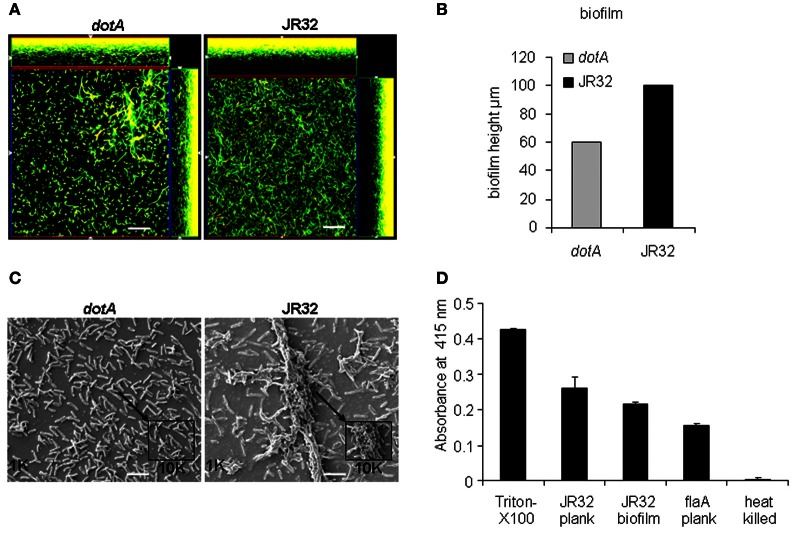
**Dot/Icm type IV secretion promotes robust *L. pneumophila* biofilm formation. (A)** Representative images showing the Live/Dead staining of WT *L. pneumophila* biofilm (top) or the type IV secretion mutant (*dotA*). Images were captured using inverted confocal Zeiss LSM 510 META microscope with a 63× water objective. Z-stacks were captured every 1 μm. Red stain indicates dead bacteria while green indicates live bacteria, scale = 10 μm. **(B)** Representation of biofilm height in μm. **(C)** Scanning electron microscopy (SEM) of JR32 and *dotA* mutant. Larger images were captured with the 1000× objective lens while smaller images were magnified 10,000×, scale = 10 μm. **(D)** Pore-forming activity of *L. pneumophila* as determined by contact-dependent hemolysis of sheep red blood cells (sRBC) and measured at *A*_415_ nm. Data are presented as means ± SD of two independent experiments each performed in quadruplicates.

The *dotA* mutant, which lacks a functional type IV secretion system, produced a thinner and less filamentous biofilm (Figure 1A) that was 60 μm high (Figure 1B). The homogenous green color, with lack of red coloring indicates that the constituent bacteria are viable. This result suggests that a functional Dot/Icm type IV secretion system promotes biofilm formation.

To confirm that *L. pneumophila* is able to form biofilm *in vitro* and this process is dependent upon type IV secretion system, we examined biofilm formation using scanning electron microscopy (SEM). Our data showed that JR32 strain formed a robust biofilm with characteristic towers whereas the *dotA* mutant failed to do so (Figure 1C). These data confirm that robust biofilm formation requires type IV secretion system.

The pore forming activity of *L. pneumophila* has been shown to contribute to macrophage cytotoxicity and requires a functional type IV secretion system (Kirby et al., 1998; Alli et al., 2000). To examine whether biofilm-derived *L. pneumophila* exhibit pore-forming activity, contact-dependent hemolysis of sheep red blood cells (RBCs) was performed (Kirby et al., 1998). Triton-X100 and heat-killed bacteria were used as positive and negative controls, respectively. The *flaA* mutant lacking flagellin and expresses a functional type IV secretion system was also examined (Figure 1D). Biofilm-derived and planktonic *L. pneumophila* and *flaA* mutant were capable of lysing the RBCs, suggesting that biofilm-derived *L. pneumophila* exhibit a functional type IV secretion system.

### Biofilm-derived *L. pneumophila* replicates significantly more intracellularly and induces less murine macrophage death than the planktonic bacteria

**Figure 2:**
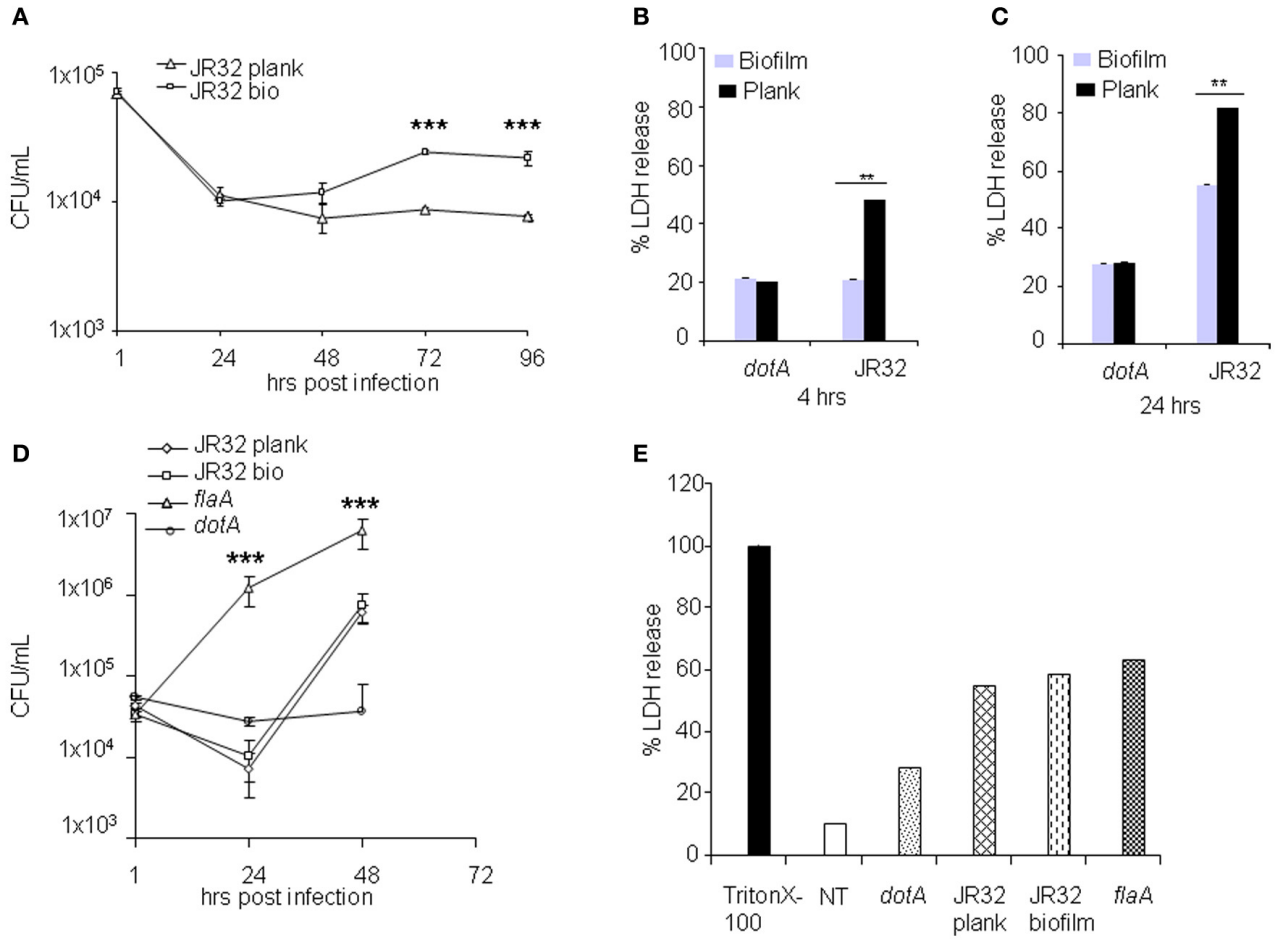
**Biofilm-derived *L. pneumophila* replicates significantly more and induces less murine macrophages death than the planktonic bacteria. (A)** BMDMs were infected with planktonic or biofilm-derived *L. pneumophila* at an MOI of 0.5. CFUs were scored at 1, 24, 48, 72, and 96 h. Data are presented as mean ± SD of two independent experiments each performed in duplicates. Asterisks indicate significant differences (^***^P < 0.001). BMDMs were not infected (NT) or infected with *L. pneumophila* JR32 (planktonic or biofilm) or the *dotA* mutant at an MOI of 0.5 for **(B)** 4 or **(C)** 24 h. The fold change in LDH release was measured from the overall population of macrophages. Data are presented as means ± SD of two independent experiments each performed in quadruplicates. Asterisks indicate significant differences (^**^P < 0.01). **(D)** The hMDMs were infected with *L. pneumophila* strain JR32 (planktonic or biofilm). CFUs were quantified at 1, 24, and 48 h post-infection. Data are representative as means ± SD of quintuplicate samples. **(E)** The hMDMs were not infected (NT) or infected with *L. pneumophila* JR32 (planktonic or biofilm), *dotA* or the *flaA* mutant at an MOI of 0.5 for 4 or 24 h. The fold change in LDH release was measured from the overall population of macrophages. Data are representative of mean ± SD of quadruplicate samples.

WT murine macrophages are restrictive to planktonic *L. pneumophila* replication. However, the murine macrophages response to biofilm-derived *L. pneumophila* is not known. Therefore, we examined the intracellular replication of biofilm-derived *L. pneumophila*. In contrast to planktonic bacteria, the biofilm-derived bacteria replicated significantly as indicated by the colony forming units (CFUs) over time (48–96 h) (Figure 2A). Macrophages phagocytozed similar numbers of biofilm-derived and planktonic *L. pneumophila* as demonstrated by the 1 h CFU counts.

Premature cell death restricts *L. pneumophila* replication within murine macrophages (Akhter et al., 2009, 2012). Macrophage death can be detected by measuring the enzymatic activity of lactate dehydrogensae (LDH) released from dead cells using a LDH cytotoxicity assay. Our data demonstrated that macrophages infected with biofilm-derived *L. pneumophila* produced less LDH at 4 and 24 h post-infection when compared to those infected with planktonic bacteria (Figures 2B,C). Biofilm-derived and planktonic *dotA* mutants led to the same extent of macrophage death. These data suggest that biofilm-derived *L. pneumophila* induced less cell death in murine macrophages than did planktonic-derived *L. pneumophila*.

### Human macrophages are permissive to biofilm-derived *L. pneumophila* as they are to planktonic cultures

In contrast to murine macrophages, human monocytes-derived macrophages (hMDMs) are permissive to planktonic *L. pneumophila* at least in part due to diminished caspase-1 and -7 activation (Horwitz, 1983; Abdelaziz et al., 2011a,b). Replication within macrophages is essential for establishing Legionnaire's pneumonia. Thus, we evaluated the intracellular growth of biofilm-derived *L. pneumophila* in hMDMs. The biofilm-derived *L. pneumophila* replicated similar to planktonic *L. pneumophila* in hMDMs (Figure 2D). As expected the *dotA* mutant did not replicate whereas *L. pneumophila* mutant lacking flagellin replicated the most (Figure 2D). This difference was not due to differential uptake since phagocytosis of all tested strains was similar as shown by the 1 h CFUs (Figure 2D).

Furthermore, we tested macrophage death by measuring percentage of LDH released after 24 h of infection. Planktonic and biofilm-derived *L. pneumophila* caused similar amount of LDH release from hMDMs while *dotA* mutant caused less cell death (Figure 2E). Human macrophages infected with the *flaA* mutant also released comparable amounts of LDH (Figure 2E). These data suggest that biofilm-derived *L. pneumophila* behave similarly to planktonic *L. pneumophila* in hMDMs.

### Biofilm-derived *L. pneumophila* avoids caspase-1 and -7 activation in murine macrophages due to lack of flagellin expression

**Figure 3:**
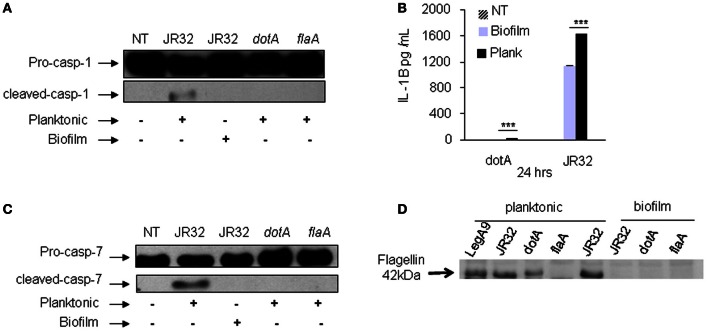
**Biofilm-derived *L. pneumophila* did not promote caspase-1 or 7 activation in murine macrophages and showed significantly less IL-1β release due to lack of flagellin expression. (A)** Pro and active caspase-1 were detected in cell extracts using caspase-1 antibody. WT BMDMs were either not treated (NT) or infected with *L. pneumophila* JR32 (biofilm or planktonic), the *dotA* or the *flaA* mutant for 2 h. **(B)** The amount of IL-1β was determined in supernatants of WT infected with JR32 (biofilm or planktonic) or the *dotA* mutant after 24 h. Data are presented as means ± SD of one experiment performed in quadruplicate. Asterisks indicate significant differences (^***^P < 0.001). **(C)** Activation of caspase-7 was detected in cell extracts using caspase-7 antibody. **(D)** Western blot analysis of planktonic and biofilm-derived *L. pneumophila* with flagellin antibody.

Upon detection of bacterial flagellin by Nlrc4 and Naip5, WT murine macrophages restrict planktonic *L. pneumophila* replication via caspase-1 and -7 activation. Caspase-7 promotes the fusion of the *L. pneumophila*-containing vacuole with the lysosome and bacterial degradation whereas caspase-1 contributes to pyroptosis and IL-1β release (Akhter et al., 2009, 2012). Because biofilm-derived *L. pneumophila* replicate more efficiently in murine macrophages and exhibited less cell death when compared to planktonic, we tested whether mouse macrophages activated caspase-1 in response to biofilm-derived bacteria. The *dotA* and *flaA* mutants were used as negative controls since they both avoid caspase-1 activation. In contrast to planktonic bacteria, biofilm-derived *L. pneumophila* did not promote caspase-1 activation as denoted by the detection of the cleaved active band by western blot (Figure 3A). These data indicate that murine macrophages respond to biofilm-derived *L. pneumophila* differentially than to planktonic *L. pneumophila.*

IL-1β maturation is promoted by active caspase-1 in WT macrophages infected with planktonic *L. pneumophila*. Therefore, we tested IL-1β release in culture supernatants from murine macrophages infected with planktonic, biofilm-derived *L. pneumophila* and the *dotA* mutant. Our data demonstrate that murine macrophages infected with biofilm-derived bacteria released 30% less IL-1β compared to that released after planktonic *L. pneumophila* infection (Figure 3B). This result indicates that the inflammatory response to biofilm-derived *L. pneumophila* in murine macrophages is less than that elicited in response to planktonic bacteria.

During infection of planktonic *L. pneumophila*, murine macrophages activate caspase-7 via the inflammasome complex contributing to bacterial restriction (Akhter et al., 2009, 2012), but this response has not been characterized in biofilm-derived *L. pneumophila*. Therefore, we tested caspase-7 activation of murine macrophages in response to biofilm-derived and planktonic *L. pneumophila*. In contrast to planktonic bacteria, biofilm-derived *L. pneumophila* did not promote caspase-7 cleavage (Figure 3C), indicating that biofilm-derived bacteria do not elicit caspase-7 activation, thereby allowing them to evade a restrictive mechanism employed by murine macrophages.

Flagellin mediates restriction of *L. pneumophila* in murine macrophages and the *flaA* mutant has been shown to replicate significantly more than the parent strain (Amer et al., 2006). Since biofilm-derived bacteria replicated significantly in murine macrophages and failed to activate caspase-1 or 7, we hypothesized that biofilm-derived bacteria down regulates flagellin expression. Western blot analysis of bacterial lysates using specific flagellin antibodies demonstrated that biofilm-derived bacteria diminished flagellin expression compared to planktonic bacteria (Figure 3D). Collectively, these results indicate that biofilm *L. pneumophila* do not activate the inflammasome because of lack of flagellin expression compared to planktonic *L. pneumophila*.

### Phagosomes containing biofilm-derived *L. pneumophila* evade fusion with the lysosomes

**Figure 4:**
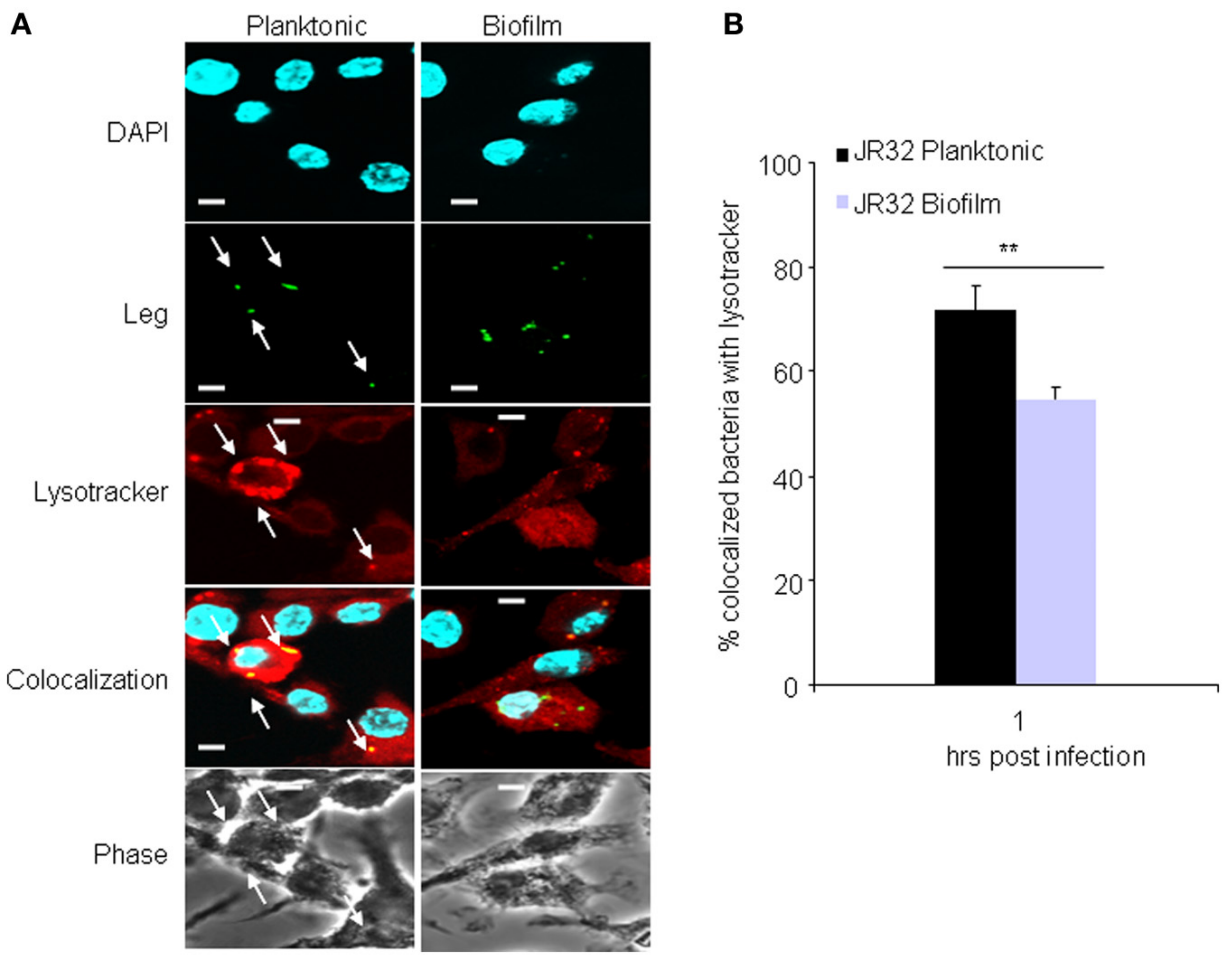
**Vacuoles harboring biofilm-derived *L. pneumophila* bacteria significantly evade fusion with lysosomes. (A)** Representative images of WT BMDMs infected for 1h with JR32 planktonic or biofilm. Nuclei are stained blue with DAPI and *L. pneumophila* stained green with *L. pneumophila*-specific antibody. Lyso-tracker red was used to stain acidified lysosomes. White arrows indicate *L. pneumophila* colocalization with lysotracker. **(B)** Percent colocalization of *L. pneumophila* with lysotracker. Images were captured with the 60× objective and magnified 3×, scale bar = 10 μm. Data are presented as means ± SD of three independent experiments each performed in triplicates. Asterisks indicate significant difference (^**^P < 0.01).

In murine macrophages, *L. pneumophila* replication is restricted by caspase-1 and -7 activation that result in phagosome-lysosome fusion, promoting bacterial degradation (Akhter et al., 2009, 2012). We examined the colocalization of planktonic and biofilm-derived bacteria with lysosomes at 1 h post-infection. Approximately 55% of biofilm-derived *L. pneumophila* resided in lysosomes (Figures 4A,B). Yet, 72% of planktonic bacteria resided in lysosomes. These results suggests that significantly more biofilm-derived *L. pneumophila* evade lysosomal degradation in macrophages allowing the bacteria to survive within the host and replicate as indicated by increased CFUs in (Figure 4A).

## Discussion

Legionnaire's disease is a severe pneumonia that infects the elderly and the immune compromised. There is no human to human transmission whereas infection occurs by inhalation of contaminated droplets from biofilms lining air conditioners and fresh water fountains. A biofilm is a highly-organized, multicellular community affixed to an inert or biological surface and is the preferred lifestyle of most bacteria. Bacterial populations within a biofilm, as opposed to their planktonic counterparts, are highly resistant to eradication (Flemming and Wingender, 2010). While some pathogens form biofilms within infected organs to resist phagocytosis and immune responses, others such as *L. pneumophila* form biofilms in nature and in medical and dental devices to serve as sources of outbreaks of Legionnaire's disease. In this case, the biofilm setting serves as niche where the bacterium is recalcitrant to most antibacterial agents (Rogers et al., 1994; Brown and Barker, 1999; Lau and Ashbolt, 2009).

In this study we reproduced *L. pneumophila* biofilm formation *in vitro*. Since infection occurs through the inhalation of *L. pneumophila*-containing droplets, we also simulated the dispersion of biofilm by vortex. Because once inhaled, the organism adapts an intracellular niche within macrophages, we examined the behavior of biofilm-derived *L. pneumophila* within murine and human macrophages.

We showed that biofilm-derived bacteria are more filamentous than planktonic bacteria (Figure 1A). This characteristic may be due to adaptation of the bacteria to a harsh environment providing an energy saving benefit for survival. Alternatively, the filamentous multinucleated characteristic may facilitate rapid division into planktonic form when encountering a parasitic host (Taylor et al., 2009). Notably, macrophages derived from mice and human donors phagocytozed equivalent numbers of biofilm-derived and planktonic organisms as demonstrated at 1 h post infection. Therefore, the filamentous phenotype of *L. pneumophila* within the biofilm did not impact their phagocytosis.

We demonstrated that robust biofilm formation by *L. pneumophila* is promoted by the DotA type IV secretion system. This finding corroborates several studies demonstrating the contribution of secreted bacterial molecules necessary for biofilm formation (Taylor et al., 2009). Secreted bacterial factors are utilized for quorum-sensing, biofilm building, and twitching motility during biofilm formation (Taylor et al., 2009). Therefore, identification and characterization of bacterial factors that are required for *L. pneumophila* biofilm formation can be targeted to prevent or eradicate biofilm formation and transmission of Legionnaires' disease in medical and industrial settings. Furthermore, *L. pneumophila* embedded within multispecies biofilms may respond to signal molecules produced by other bacteria promoting us to rethink current *L. pneumophila* research paradigms (Taylor et al., 2009).

Although the innate immune response of macrophages to planktonic *L. pneumophila* is under investigation by many scientists, the macrophage response to biofilm-derived *L. pneumophila* is yet to be elucidated. In this study, we found that biofilm-derived *L. pneumophila* replicate significantly more in murine macrophages than do planktonic bacteria. This result suggests that studies employing planktonic *L. pneumophila* in murine macrophages may not recapitulate the biofilm-derived mode of infection in humans. Yet, hMDM responded similarly to biofilm-derived and planktonic *L. pneumophila* establishing more validation for human based studies whether using planktonic or biofilm-derived *L. pneumophila*.

For an unidentified reason, planktonic flagellated *L. pneumophila* escapes detection by human NAIP and NLRC4 contributing to the permissiveness of human macrophages to the pathogen (Abdelaziz et al., 2011a,b; Ge et al., 2012). Studies by the Shao group indicated that human NAIP does not appear to respond to *L. pneumophila* flagellin (Ge et al., 2012). We also showed that human NLRC4 does not respond to flagellated *L. pneumophila* (Abdelaziz et al., 2011a,b). Yet, human NLRC4 activates caspase-1 in response to flagellated *Salmonella* (Abdelaziz et al., 2011b). Here we found that biofilm-derived *L. pneumophila* that seems to diminish flagellin expression replicates to the same extent as planktonic cultures in human macrophages. Since *L. pneumophila* flagellin detection is nonetheless avoided in human cells, it is plausible to expect that the *flaA* mutant would replicate to the same extent, however, this was not the case. The planktonic *flaA* mutant replicated more that the parent strain whether planktonic or biofilm-derived. This result suggests that biofilm-derived *L. pneumophila* may still express scarce amounts of flagellin contributing to its modest restriction via a caspase-1 independent mechanism. This remains to be elucidated by further studies.

Unlike planktonic *L. pneumophila*, biofilm-derived *L. pneumophila* do not activate caspase-1 and -7 in murine macrophages. The activation of caspase-1 and -7 promote the fusion of vacuoles containing *L. pneumophila* with lysosomes and bacterial degradation (Amer et al., 2006; Akhter et al., 2009; Franchi et al., 2009). Thus, our results suggest that biofilm derived *L. pneumophila* evades phagosome-lysosome fusion by avoiding caspase-1 and -7 activation. Macrophages infected with biofilm-derived bacteria exhibited significantly less IL-1β release and macrophage death compared to those infected with planktonic bacteria. IL-1β is a pro-inflammatory cytokine activated by caspase-1. Thus, by reducing IL-1β release, biofilm-derived *L. pneumophila* avoids major inflammatory responses and the recruitment of inflammatory cells to the infected lungs.

A better understanding of the innate immune response to biofilm-derived *L. pneumophila* will pave the way for the development of novel diagnosis and treatment strategies.

### Author contributions

Arwa Abu Khweek designed and performed the experiments, analyzed the results, and wrote the manuscript. Natalia S. Fernández Dávila, Kyle Caution, Anwari Akhter, Basant A. Abdulrahman, Mia Tazi, Hoda Hassan, Laura A. Novotny, Lauren O. Bakaletz contributed to the performance of the experiments and editing of the manuscript. Amal O. Amer helped in the design of the experiments, interpretation of the results, and editing of the manuscript.

### Conflict of interest statement

The authors declare that the research was conducted in the absence of any commercial or financial relationships that could be construed as a potential conflict of interest.

## References

[B1] Abdelaziz D. H. Gavrilin M. A. Akhter A. Caution K. Kotrange S. Khweek A. A. (2011a). Apoptosis-associated speck-like protein (ASC) controls *Legionella pneumophila* infection in human monocytes. J. Biol. Chem. 286, 3203–3208 10.1074/jbc.M110.197681 21097506 PMC3030324

[B2] Abdelaziz D. H. Gavrilin M. A. Akhter A. Caution K. Kotrange S. Khweek A. A. (2011b). Asc-dependent and independent mechanisms contribute to restriction of *Legionella pneumophila* infection in murine macrophages. Front. Microbiol. 2:18 10.3389/fmicb.2011.00018 21713115 PMC3112328

[B3] Abdulrahman B. A. Khweek A. A. Akhter A. Caution K. Kotrange S. Abdelaziz D. H. (2011). Autophagy stimulation by rapamycin suppresses lung inflammation and infection by *Burkholderia cenocepacia* in a model of cystic fibrosis. Autophagy 7, 1359–1370 10.4161/auto.7.11.17660 21997369 PMC3359483

[B4] Abu Kwaik Y. Eisenstein B. I. Engleberg N. C. (1993). Phenotypic modulation by *Legionella pneumophila* upon infection of macrophages. Infect. Immun. 61, 1320–1329 8454334 10.1128/iai.61.4.1320-1329.1993 PMC281365

[B5] Akhter A. Caution K. Abu Khweek A. Tazi M. Abdulrahman B. A. Abdelaziz D. H. (2012). Caspase-11 promotes the fusion of phagosomes harboring pathogenic bacteria with lysosomes by modulating actin polymerization. Immunity 37, 35–47 10.1016/j.immuni.2012.05.001 22658523 PMC3408798

[B6] Akhter A. Gavrilin M. A. Frantz L. Washington S. Ditty C. Limoli D. (2009). Caspase-7 activation by the Nlrc4/Ipaf inflammasome restricts *Legionella pneumophila* infection. PLoS Pathog. 5:e1000361 10.1371/journal.ppat.1000361 19343209 PMC2657210

[B7] Al-Khodor S. Price C. T. Habyarimana F. Kalia A. Abu Kwaik Y. (2008). A Dot/Icm-translocated ankyrin protein of *Legionella pneumophila* is required for intracellular proliferation within human macrophages and protozoa. Mol. Microbiol. 70, 908–923 10.1111/j.1365-2958.2008.06453.x 18811729 PMC3064707

[B8] Alli O. A. Gao L. Y. Pedersen L. L. Zink S. Radulic M. Doric M. (2000). Temporal pore formation-mediated egress from macrophages and alveolar epithelial cells by *Legionella pneumophila*. Infect. Immun. 68, 6431–6440 10.1128/IAI.68.11.6431-6440.2000 11035756 PMC97730

[B9] Amer A. O. (2010). Modulation of caspases and their non-apoptotic functions by *Legionella pneumophila*. Cell. Microbiol. 12, 140–147 10.1111/j.1462-5822.2009.01401.x 19863553

[B10] Amer A. Franchi L. Kanneganti T. D. Body-Malapel M. Ozoren N. Brady G. (2006). Regulation of Legionella phagosome maturation and infection through flagellin and host Ipaf. J. Biol. Chem. 281, 35217–35223 10.1074/jbc.M604933200 16984919

[B11] Atlas R. M. (1999). Legionella: from environmental habitats to disease pathology, detection and control. Environ. Microbiol. 1, 283–293 10.1046/j.1462-2920.1999.00046.x 11207747

[B12] Brown M. R. Barker J. (1999). Unexplored reservoirs of pathogenic bacteria: protozoa and biofilms. Trends Microbiol. 7, 46–50 10068997 10.1016/s0966-842x(98)01425-5

[B13] Coers J. Kagan J. C. Matthews M. Nagai H. Zuckman D. M. Roy C. R. (2000). Identification of Icm protein complexes that play distinct roles in the biogenesis of an organelle permissive for *Legionella pneumophila* intracellular growth. Mol. Microbiol. 38, 719–736 10.1046/j.1365-2958.2000.02176.x 11115108

[B14] Costerton J. W. Geesey G. G. Cheng K. J. (1978). How bacteria stick. Sci. Am. 238, 86–95 635520 10.1038/scientificamerican0178-86

[B15] Donlan R. M. Forster T. Murga R. Brown E. Lucas C. Carpenter J. (2005). *Legionella pneumophila* associated with the protozoan *Hartmannella vermiformis* in a model multi-species biofilm has reduced susceptibility to disinfectants. Biofouling 21, 1–7 10.1080/08927010500044286 16019386

[B16] Flemming H. C. Wingender J. (2010). The biofilm matrix. Nat. Rev. Microbiol. 8, 623–633 10.1038/nrmicro2415 20676145

[B17] Fliermans C. B. Cherry W. B. Orrison L. H. Smith S. J. Tison D. L. Pope D. H. (1981). Ecological distribution of *Legionella pneumophila*. Appl. Environ. Microbiol. 41, 9–16 7013702 10.1128/aem.41.1.9-16.1981 PMC243633

[B18] Franchi L. Eigenbrod T. Munoz-Planillo R. Nunez G. (2009). The inflammasome: a caspase-1-activation platform that regulates immune responses and disease pathogenesis. Nat. Immunol. 10, 241–247 10.1038/ni.1703 19221555 PMC2820724

[B19] Fraser D. W. Deubner D. C. Hill D. L. Gilliam D. K. (1979). Nonpneumonic, short-incubation-period Legionellosis (Pontiac fever) in men who cleaned a steam turbine condenser. Science 205, 690–691 10.1126/science.462175 462175

[B20] Ge J. Gong Y. N. Xu Y. Shao F. (2012). Preventing bacterial DNA release and absent in melanoma 2 inflammasome activation by a Legionella effector functioning in membrane trafficking. Proc. Natl. Acad. Sci. U.S.A. 109, 6193–6198 10.1073/pnas.1117490109 22474394 PMC3341053

[B21] Harb O. S. Abu Kwaik Y. (2000). Essential role for the *Legionella pneumophila* rep helicase homologue in intracellular infection of mammalian cells. Infect. Immun. 68, 6970–6978 11083821 10.1128/iai.68.12.6970-6978.2000 PMC97806

[B22] Horwitz M. A. (1983). The Legionnaires' disease bacterium (*Legionella pneumophila*) inhibits phagosome-lysosome fusion in human monocytes. J. Exp. Med. 158, 2108–2126 6644240 10.1084/jem.158.6.2108 PMC2187157

[B23] Horwitz M. A. Silverstein S. C. (1980). Legionnaires' disease bacterium (*Legionella pneumophila*) multiples intracellularly in human monocytes. J. Clin. Invest. 66, 441–450 10.1172/JCI109874 7190579 PMC371671

[B24] Isberg R. R. O'Connor T. J. Heidtman M. (2009). The *Legionella pneumophila* replication vacuole: making a cosy niche inside host cells. Nat. Rev. Microbiol. 7, 13–24 10.1038/nrmicro1967 19011659 PMC2631402

[B25] Kirby J. E. Vogel J. P. Andrews H. L. Isberg R. R. (1998). Evidence for pore-forming ability by *Legionella pneumophila*. Mol. Microbiol. 27, 323–336 10.1046/j.1365-2958.1998.00680.x 9484888

[B26] Lau H. Y. Ashbolt N. J. (2009). The role of biofilms and protozoa in Legionella pathogenesis: implications for drinking water. J. Appl. Microbiol. 107, 368–378 10.1111/j.1365-2672.2009.04208.x 19302312

[B27] Lettinga K. D. Verbon A. Weverling G. J. Schellekens J. F. Den Boer J. W. Yzerman E. P. (2002). Legionnaires' disease at a Dutch flower show: prognostic factors and impact of therapy. Emerg. Infect. Dis. 8, 1448–1454 10.3201/eid0812.020035 12498662 PMC2738521

[B28] Marston B. J. Plouffe J. F. File T. M. Jr. Hackman B. A. Salstrom S. J. Lipman H. B. (1997). Incidence of community-acquired pneumonia requiring hospitalization. Results of a population-based active surveillance Study in Ohio. The Community-Based Pneumonia Incidence Study Group. Arch. Intern. Med. 157, 1709–1718 9250232

[B29] Rogers J. Dowsett A. B. Dennis P. J. Lee J. V. Keevil C. W. (1994). Influence of temperature and plumbing material selection on biofilm formation and growth of *Legionella pneumophila* in a model potable water system containing complex microbial flora. Appl. Environ. Microbiol. 60, 1585–1592 8017938 10.1128/aem.60.5.1585-1592.1994 PMC201521

[B30] Roy C. R. (2002). The Dot/lcm transporter of *Legionella pneumophila*: a bacterial conductor of vesicle trafficking that orchestrates the establishment of a replicative organelle in eukaryotic hosts. Int. J. Med. Microbiol. 291, 463–467 11890545 10.1078/1438-4221-00154

[B31] Santic M. Molmeret M. Abu Kwaik Y. (2005). Maturation of the *Legionella pneumophila*-containing phagosome into a phagolysosome within gamma interferon-activated macrophages. Infect. Immun. 73, 3166–3171 10.1128/IAI.73.5.3166-3171.2005 15845527 PMC1087382

[B32] Sethi K. K. Brandis H. (1983). Direct demonstration and isolation of *Legionella pneumophila* (serogroup 1) from bathroom water specimens in a hotel. Zentralbl. Bakteriol. Mikrobiol. Hyg. B 177, 402–405 6367308

[B33] Spitalny K. C. Vogt R. L. Orciari L. A. Witherell L. E. Etkind P. Novick L. F. (1984). Pontiac fever associated with a whirlpool spa. Am. J. Epidemiol. 120, 809–817 6391156 10.1093/oxfordjournals.aje.a113953

[B34] Taylor M. Ross K. Bentham R. (2009). Legionella, protozoa, and biofilms: interactions within complex microbial systems. Microb. Ecol. 58, 538–547 10.1007/s00248-009-9514-z 19365668

